# The Role of Cosolvent–Water Interactions in Effects of the Media on Functionality of Enzymes: A Case Study of *Photobacterium leiognathi* Luciferase

**DOI:** 10.3390/life13061384

**Published:** 2023-06-13

**Authors:** Albert E. Lisitsa, Lev A. Sukovatyi, Anna A. Deeva, Dmitry V. Gulnov, Elena N. Esimbekova, Valentina A. Kratasyuk, Elena V. Nemtseva

**Affiliations:** 1 Biophysics Department, Siberian Federal University, 660041 Krasnoyarsk, Russia; lsukovatyy@sfu-kras.ru (L.A.S.); adeeva@sfu-kras.ru (A.A.D.); dgulnov@sfu-kras.ru (D.V.G.); enemtseva@sfu-kras.ru (E.V.N.); 2 Institute of Biophysics, Siberian Branch of Russian Academy of Sciences, 660036 Krasnoyarsk, Russia

**Keywords:** bacterial luciferase, viscosity, macromolecular crowding, water activity, non-steady-state reaction kinetics, diffusion limitation, bioluminescence, preferential interaction, enzymatic catalysis

## Abstract

**Simple Summary:**

Traditionally, enzymes have been studied in standardized buffers, but in real life, inside cells, they have to function under conditions far from “ideal”. The intracellular milieu is crowded by numerous molecules with various physical and chemical properties. What consequences does it have for enzymatic reactions? How do enzymes deal with disturbing neighbors? To examine this issue, we studied the mechanisms of influence of seven model substances with different molecular sizes on the reaction catalyzed by bacterial luciferase. This reaction passes through several stages and ends with light emission. We analyzed the responses rate in different stages to the addition of alcohols, saccharides, and biopolymers and found the counteraction of two effects. On the one hand, increased viscosity slows down many of the stages, both associative and dissociative. On the other hand, it is accompanied by an increase in the catalytic constant of luciferase, which compensates for kinetic disadvantages. We assumed that both effects could be caused by the same property of the added substances—their ability to interact with water. Our findings serve not only to shed light on the organization of the cellular metabolism, but also to improve enzyme-based biotechnologies using the recipes of nature.

**Abstract:**

A complex heterogeneous intracellular environment seems to affect enzymatic catalysis by changing the mobility of biomolecules, their stability, and their conformational states, as well as by facilitating or hindering continuously occurring interactions. The evaluation and description of the influence of the cytoplasmic matrix components on enzymatic activity are problems that remain unsolved. In this work, we aimed to determine the mechanisms of action of two-component media with cosolvents of various molecular sizes on the complex multi-stage bioluminescent reaction catalyzed by bacterial luciferase. Kinetic and structural effects of ethylene glycol, glycerol, sorbitol, glucose, sucrose, dextran, and polyethylene glycol on bacterial luciferase were studied using stopped-flow and fluorescence spectroscopy techniques and molecular dynamics simulations. We have found that diffusion limitations in the presence of cosolvents promote the stabilization of flavin substrate and peroxyflavin intermediate of the reaction, but do not provide any advantages in bioluminescence quantum yield, because substrate binding is slowed down as well. The catalytic constant of bacterial luciferase has been found to be viscosity-independent and correlated with parameters of water–cosolvent interactions (Norrish constant, van der Waals interaction energies). Crowding agents, in contrast to low-molecular-weight cosolvents, had little effect on peroxyflavin intermediate decay and enzyme catalytic constant. We attributed specific kinetic effects to the preferential interaction of the cosolvents with enzyme surface and their penetration into the active site.

## 1. Introduction

Whether in a test tube or cytosol, enzymes have to deal with the rules of the surrounding environment while their functioning story unfolds. Inside the cells, biochemical processes take place in the presence of a large number of molecules of different sizes and chemical natures which, even without directly interacting with enzymes and their substrates, could essentially influence the efficiency of biological catalysis [1,2,3,4]. To study this influence with in vitro model systems, which are usually enzymes in buffer–cosolvent mixtures, several approaches are used, depending on the main mechanism(s) addressed. Among them, Kramers’ theory and the effect of excluded volume are probably the most frequently applied concepts in this field in recent decades (see details in reviews [5,6]). Kramers’ theory considers the dependence of the rate of a (bio)chemical reaction on the viscosity of the reactant’s environment [7]. Strong dependence of the catalytic constant of the enzyme on viscosity indicates significant rearrangement of the protein structure, accompanying the chemical reaction at the active site [8]. In turn, the thermodynamic effect of excluded volume, which is caused by the presence of numerous large molecules around the protein (or crowding effect), modulates the hydrodynamic volume of macromolecules. It affects the compactness of the protein molecules, their shape and structure, conformational stability, association state, and, as a consequence, their functionality [9]. However, in addition to the alteration of intensive (bulk) properties of the environment (i.e., viscosity, ionic strength, polarity), cosolvents can participate in specific intermolecular interactions with reactants (i.e., hydrogen bonding) and change their conformations or other features.

**Figure 1:**
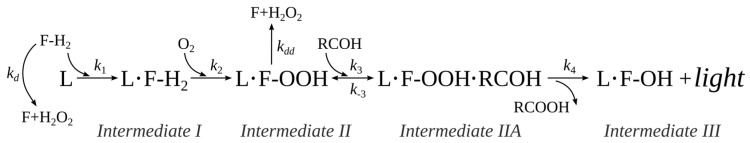
The stages of the reaction catalyzed by bacterial luciferase. L—luciferase, F—flavin mononucleotide, O_2_—molecular oxygen, RCOH and RCOOH—long-chain aldehyde and corresponding carboxylic acid, H_2_O_2_—hydrogen peroxide.

In the current study, we focus on the mechanisms of effects produced by the media on the reaction catalyzed by bacterial luciferase. In nature, luciferases are responsible for bioluminescence, i.e., for the emission of visible light by living organisms [10]. Despite the common name, luciferases from different groups of organisms have distinct structures and utilize dissimilar substrates [11]. We expected that bacterial luciferase has a more pronounced response to changes in the medium viscosity than other luciferases because the accumulation of viscogenic molecules for adaptation to adverse environmental conditions is a typical mechanism for unicellular organisms, while multicellular organisms use more complex regulation [12]. Additionally, in vitro, the reaction catalyzed by bacterial luciferase demonstrates rather low efficiency (quantum yield of bioluminescence is about 10%) [10], indicating numerous “dark” pathways accompanying the main “light” reaction pathway. This could mean that the best conditions for this reaction in vitro have not been found yet, and mimicking intracellular conditions could help approach them. Bacterial luciferase is a flavin-dependent monooxygenase utilizing reduced flavin mononucleotide, long-chain aldehyde, and oxygen as substrates [13]. The reaction proceeds through formation of at least four reaction intermediates and produces a light quantum as one of the products (Figure 1). Due to the chemical instability of the substrate, reduced flavin mononucleotide, light emission in this reaction exhibits complex non-steady-state kinetics, which reflects changes in the rate-limiting stages during the reaction time course [14]. Bacterial luciferases are routinely used for sensing and imaging in biomedicine and environmental monitoring [15,16]. Thus, understanding the mechanisms of modulation of the luciferase function by the media is needed for practical applications as well.

**Figure 2:**
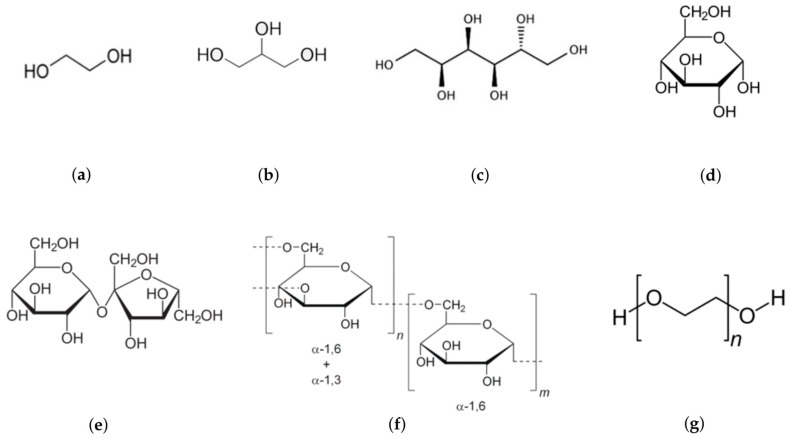
Chemical structures of the cosolvents used in the study: (**a**) ethylene glycol (62 Da), (**b**) glycerol (92 Da), (**c**) sorbitol (182 Da), (**d**) glucose (180 Da), (**e**) sucrose (342 Da), (**f**) dextran (70 kDa), (**g**) polyethylene glycol (4 kDa).

In our previous studies, we found that viscous media with glycerol and sucrose slow down both ”dark” and substrate-binding stages of the reaction catalyzed by bacterial luciferase in a diffusion-control manner [17,18,19]. Additionally, we observed an increase in the catalytic constant in the presence of sucrose [19]. However, based on just two cosolvents, it is impossible to distinguish between the effects of intensive (bulk) properties of media and specific chemical properties of the cosolvents. Therefore, to establish patterns that are more general, the range of cosolvents was expanded. In the current work, we analyzed effects of small-molecule cosolvents—ethylene glycol, glucose, and sorbitol—and polymeric additives (crowding agents)—polyethylene glycol and dextran (Figure 2). Only based on that, we could compare the general effects of polyhydric alcohols and saccharides, search for the macromolecular crowding effect manifestation, and analyze the correlations of the rate constants with physicochemical properties of the cosolvents.

Thus, the aim of this study was to reveal the mechanisms underlying the effects of cosolvents of various molecular sizes on the catalytic constant of bacterial luciferase. For this, we used mathematical modeling of the non-steady-state kinetics to obtain the rate constants of the elementary reaction stages and analyzed their viscosity dependencies in media with cosolvents. Additionally, we applied molecular dynamics simulations to estimate the contribution of protein structure change and protein–cosolvent interactions to the observed kinetic effects. That approach served to distinguish between diffusion-dependent and diffusion-independent stages and revealed the role of water–cosolvent interaction in the modulation of bacterial luciferase functionality.

## 2. Results

### 2.1. Kinetics of Bacterial Bioluminescent Reaction in Viscous Media with Different Cosolvents

Using the experimental approach described in detail elsewhere [19], we measured the non-steady-state kinetics of the bioluminescent reaction catalyzed by *Photobacterium leiognathi* luciferase in viscous solutions with different concentrations of ethylene glycol, glucose, sorbitol, dextran, and polyethylene glycol. Briefly, the flash-like kinetic curves of bioluminescence after rapid mixing of bacterial luciferase with photoreduced FMN and decanal were measured using the stopped flow technique. Bacterial luciferase under such conditions can make only one turn, since unbound reduced flavin is all oxidized by dissolved oxygen.

**Figure 3:**
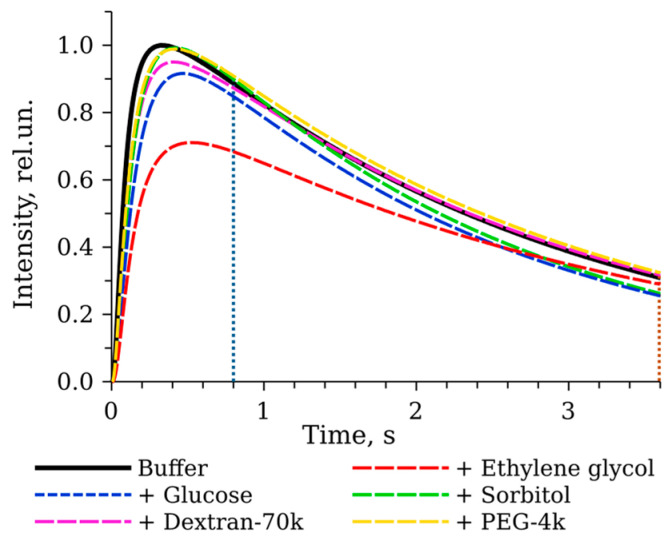
Kinetics of the bioluminescent reaction catalyzed by *P. leiognathi* luciferase in viscous solutions with different cosolvents: ethylene glycol 30 wt % (2.1 cP), glucose 20 wt % (1.95 cP), sorbitol 20 wt % (1.95 cP), Dextran-70k 10 wt % (2.48 cP), and PEG-4k 5 wt % (2.9 cP). The kinetics in buffer without additives (1 cP) is shown by solid black line for comparison. The concentrations were: luciferase—1 μM, FMNH_2_—15 μM, decanal—50 μM. Vertical dotted lines denote the approximate time range for calculating the index of the exponential decay of intensity (k_decay_).

Figure 3 shows an example of kinetic curves in solutions with viscosity of about 2 cP in the presence of different cosolvents. In viscous solutions, the kinetics of the first phase of the reaction slightly slows down compared to that in the buffer. Additionally, the decay rates in glucose and sorbitol solutions increase and the peak intensity decreases in the presence of ethylene glycol, while polymeric cosolvents seem to have no effect on the decay rate. Thus, viscosity is not the only factor determining reaction kinetics, and the effects of the medium depend on the cosolvent used.

**Figure 4:**
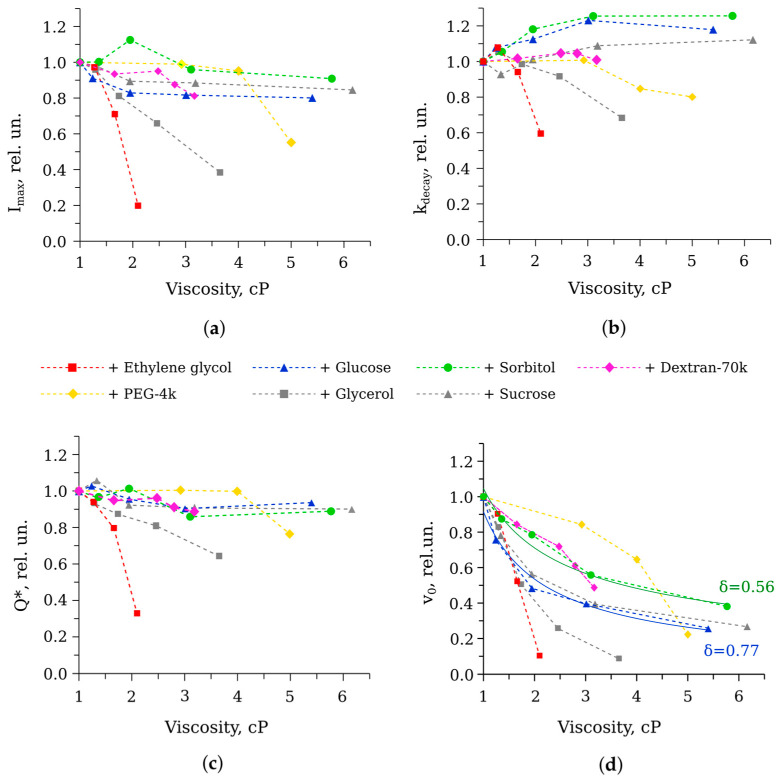
The dependence of the kinetic parameters of the reaction catalyzed by *P. leiognathi* luciferase on solution viscosity in the presence of different cosolvents: (**a**) the peak intensity (I_max_), (**b**) the decay constant (k_decay_), (**c**) the total quantum yield (Q*), (**d**) the initial velocity (v_0_). Grey markers show the previously obtained data for glycerol and sucrose solutions [19]. Aldehyde concentration was 50 μM. Dashed lines are to guide the eyes. In (**d**), solid lines show the approximations of the experimental data with equation v_0_ = A·η^−δ^ for sorbitol and glucose solutions, where A is an amplitude coefficient and η is viscosity. The corresponding index δ is indicated.

The flash-like kinetic curve of the bioluminescent reaction is usually characterized by four empirical parameters, the peak intensity (I_max_), the decay constant (k_decay_), the initial velocity (v_0_), and the total quantum yield (Q*). All of them are the results of combined rates of individual reaction stages. The methods for calculating these parameters were described in detail in our previous paper [19]. Empirical parameters of the kinetic curves obtained in the presence of different cosolvents are shown in Figure 4.

Some patterns could be seen from the viscosity dependencies (Figure 4): (i) the majority of the cosolvents only slightly changed the peak intensity, the decay rate, and, as a consequence, the total quantum yield of the reaction; only ethylene glycol and the previously studied glycerol caused a decrease in these parameters; and (ii) viscous media considerably slowed down the initial velocity of the reaction, but in a different manner; only for some cosolvents, the dependence v_0_ (η) could be well approximated by the power law function, which was a sign of diffusion limitation. Hence, the influence of the studied cosolvents on the elementary steps of the reaction catalyzed by bacterial luciferase is different, and it is not entirely due to increased viscosity.

### 2.2. The Rates of the Individual Stages of the Bacterial Bioluminescent Reaction in Media with Cosolvents

**Figure 5:**
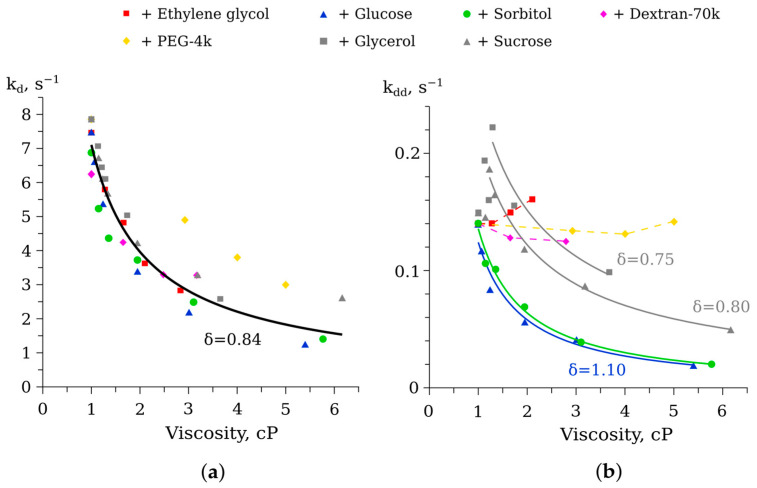
The dependence of the rate constants of FMNH_2_ autoxidation k_d_ (**a**), and the dark decay of C(4a)-hydroperoxyflavin intermediate, k_dd_ (**b**), on viscosity in solutions with different cosolvents. Gray markers show previously obtained data for glycerol and sucrose solutions [17]. Solid lines refer to the approximation of the experimental data with equation k_d,dd_ = A·η^−δ^, where A is an amplitude coefficient and η is viscosity. The corresponding indexes δ are indicated. Dashed lines are to guide the eyes.

The rates of reduced flavin oxidation by dissolved oxygen and the dark decay of peroxyflavin intermediate of the reaction (k_d_ and k_dd_ in Figure 1, respectively) in solutions of ethylene glycol, glucose, sorbitol, dextran, and polyethylene glycol were estimated in special experiments. For this, the kinetics of flavin absorbance change at 445 nm was measured, and the approximation of two time ranges with exponential functions was performed (see details in [17]). The obtained dependencies of k_d_ and k_dd_ on solution viscosity are shown in Figure 5.

The oxidation of reduced flavin by dissolved oxygen is a complex multi-stage process [20], but its rate could be characterized by the apparent constant, k_d_. We found that viscosity dependencies of k_d_ were quite uniform for the studied solutions (Figure 5a). They followed the power law function with an index of about –0.84, which is a feature of the process close to diffusion-controlled one. The only exception was polyethylene glycol, but that could be attributed to uncertainty in determination of viscosity of its solutions (i.e., possible overestimation of a viscosity value by a molecular rotor technique).

The peroxyflavin intermediate of the reaction (Intermediate II in Figure 1) could spontaneously decompose unless an aldehyde was bound to it. The change in the decay rate of Intermediate II, k_dd_, in solutions of different cosolvents is shown in Figure 5b. We observed a decrease in the rate constant in media with small-molecule cosolvents except ethylene glycol. The latter caused the peroxyflavin intermediate destabilization: k_dd_ increased by a factor of up to 1.2. The polymeric additives were found to have little effect on the decomposition of Intermediate II.

**Figure 6:**
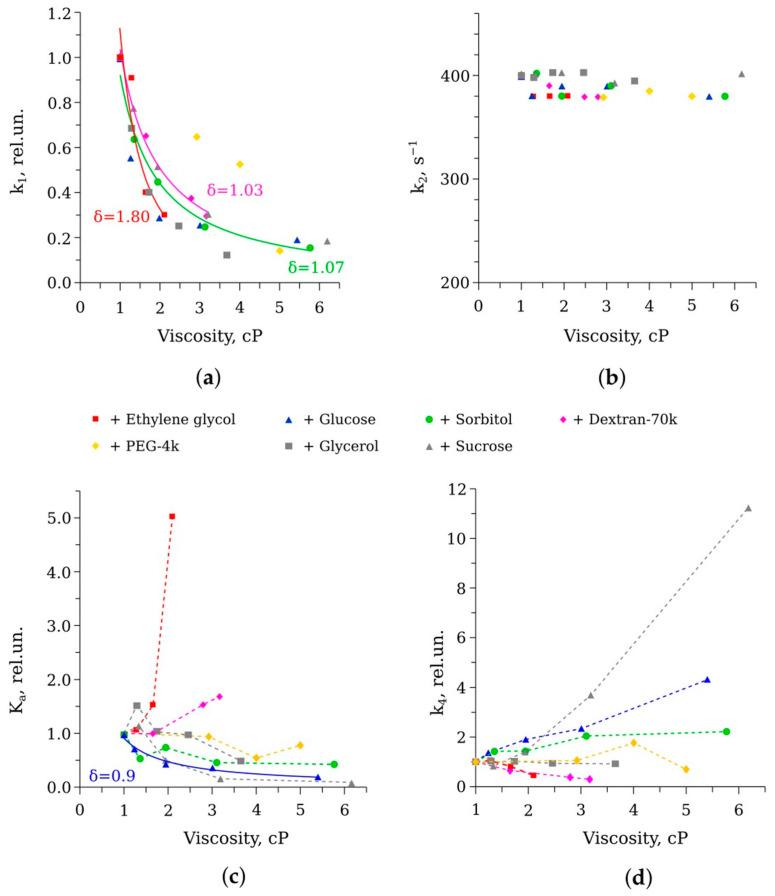
The dependence of the rate constants on media viscosity in the presence of different cosolvents: (**a**) binding constants of the reduced flavin k_1_; (**b**) binding constants of oxygen k_2_; (**c**) the association constant of aldehyde binding, K_a_ = k_3_/k_–3_; (**d**) catalytic constant k_4_. Gray markers show the previously obtained data for glycerol and sucrose solutions [19]. In (**a**,**c**,**d**), the normalized values are shown (divided by the value in the buffer with η = 1 cP). Dashed lines are to guide the eyes. Solid lines refer to the approximation of the experimental data with equation k = A·η^−δ^, where A is an amplitude coefficient and η is viscosity. The corresponding indexes δ are indicated.

For each solution, the set of five kinetic curves obtained with different decanal concentrations (10, 20, 30, 40, and 50 μM) was fitted using a special mathematical model to determine the rate constants of the individual reaction stages k_1_, k_2_, k_3_, k_–3_, and k_4_ (Figure 1). A detailed description of the mathematical modeling of bioluminescent reaction kinetics can be found in our previous paper [19]. Based on that, we analyzed how the rates of separate reaction stages depended on viscosity (Figure 6).

The following patterns were observed for different rate constants:

(i) k_1_—binding of reduced flavin or Intermediate I formation (Figure 6a).

Cosolvents such as sorbitol, glucose, sucrose, and dextran slowed down the flavin binding in a manner close to diffusion limitation (δ ≈ 1), while ethylene glycol and glycerol demonstrated the overdamped effect (δ > 1).

(ii) k_2_—binding of oxygen or Intermediate II formation (Figure 6b).

None of the cosolvents had any substantial effect on the rate of that, very fast, stage.

(iii) K_a_ = k_3_/k_–3_—binding of decanal or Intermediate IIA formation (Figure 6c).

The most pronounced decrease was observed in solutions of glucose, sorbitol, and sucrose, while in solutions of ethylene glycol and dextran there was a tendency to increase aldehyde binding.

(iv) k_4_—formation and decay of the electronically excited Intermediate III (Figure 6d).

k_4_, which could be considered a catalytic constant of luciferase, decreased in the presence of ethylene glycol and dextran. Solutions of glucose, sorbitol, and sucrose accelerated this stage, while glycerol and polyethylene glycol caused negligible effects.

Using individual rate constants, we calculated the Michaelis constants of luciferase for reduced flavin (K_M_^F^ = k_2_/k_1_) and decanal (K_M_^a^ = (k_–3_ + k_4_)/k_3_). In the buffer, the values of 1–2 μM and 25–50 μM were obtained for K_M_^F^ and K_M_^a^, respectively, which was in good agreement with the previously reported data [21]. In viscous media, the Michaelis constants changed in reverse manner to the dependencies of k_1_ and K_a_ (Figure 6a,c), indicating a decrease in luciferase affinity to the substrates in the presence of the majority of the cosolvents (data not shown).

### 2.3. Molecular Dynamics of Bacterial Luciferase in the Presence of Cosolvents

Conventionally, the mechanisms of the cosolvent effects on the catalytic constant of the enzyme can be divided into direct and indirect. The direct effect of a cosolvent could be associated with the penetration of small molecules into catalytic gorge of the enzyme, which could perturb the formation of reaction intermediates or upset the transition state of the enzyme–substrate complex. Indirect mechanisms could involve changes in the protein tertiary structure or the dynamics of its elements, which would cause reorganization of the active site. Both types of the mechanisms were studied by molecular dynamics technique.

Bacterial luciferase is a heterodimer with two very similar, but not identical, subunits with a TIM-barrel structure [22]. The α-subunit contains the active site and has an important mobile loop, which is believed to function as a lid after substrate binding [23]. Such structural organization is characteristic of the entire group of these proteins due to the high homology of their amino acid sequences [24]. However, phylogenetic analysis revealed that all known bacterial luciferases are divided into two subfamilies, which are distinct both structurally and functionally [25]. Enzymes from two subfamilies (such as *Vibrio harveyi* and *Photobacterium leiognathi*) differ slightly in the architecture of the active site gorge and in the mode of substrate binding, as well as in the composition of the functional mobile loop. Our previous research showed that they are also characterized by distinct dynamic properties [26,27]. Therefore, in the present study, the homology-modeled structure of *P. leiognathi* luciferase was used instead of the resolved crystal structure of *V. harveyi* luciferase.

We simulated the molecular dynamics of *P. leiognathi* luciferase in the presence of small-molecule cosolvents, the effects of which were studied experimentally, as described above. Those were ethylene glycol, glycerol, sorbitol, glucose, and sucrose. Using the results of the simulation, we answered the following four questions.

(i) Do the cosolvents cause a change in the luciferase tertiary structure?

**Figure 7:**
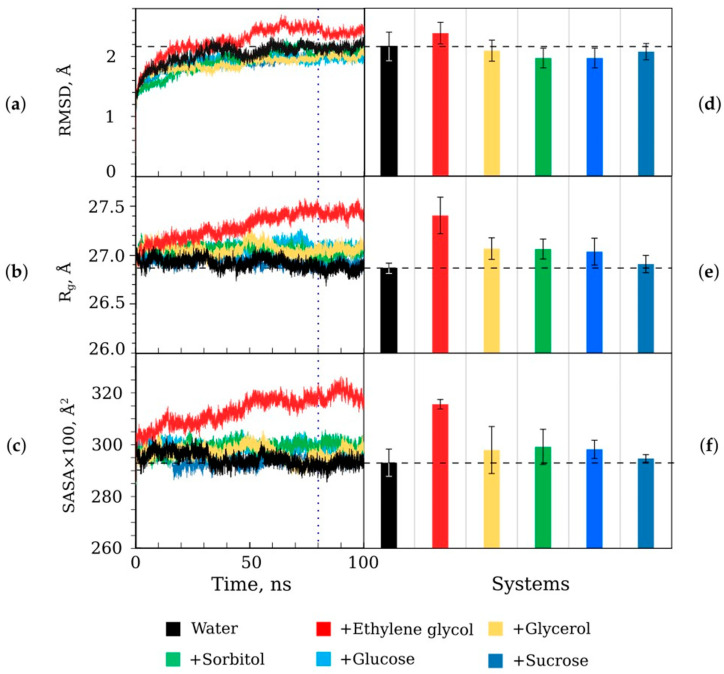
The structural parameters of *P. leiognathi* luciferase obtained using the models with water and the mixtures with ethylene glycol (30 wt %), glycerol (40 wt %), sorbitol (40 wt %), glucose (40 wt %), and sucrose (40 wt %); (**a**,**d**) the root-mean-square-deviation of C_α_-atoms (RMSD); (**b**,**e**) the gyration radius for all protein atoms (R_g_); (**c**,**f**) the total solvent accessible surface area (SASA). Panels (**a**–**c**) show parameter changes during 100 ns simulations (curves are the average for the three simulation runs (Figure S1)). Panels (**d**–**f**) show the values of the parameters for the last 20 ns of the trajectories as mean ± standard deviation. Dashed lines refer to the value in water. Dotted line indicates the range over which the parameters for (**d**–**f**) were calculated (80–100 ns).

Parameters such as the root-mean-square deviation of C_α_-atoms (RMSD), the gyration radius for all protein atoms (R_g_), and the total solvent accessible surface area (SASA) of luciferase in the presence of cosolvents was estimated (Figure 7). Ethylene glycol caused a pronounced increase in all the parameters, while sucrose had no effect. The other cosolvents slightly decreased RMSD and increased R_g_ and SASA. The observed effects could be attributed to the interaction of the cosolvent molecules (except sucrose) with the side chains of amino acids on the surface of luciferase. Since, in the presence of ethylene glycol, the RMSD of luciferase changed by <0.2 Å compared with water and we observed the stationary level of that parameter during the last 20 ns of the modeling, we excluded the denaturation of the protein by that cosolvent (Figure 7a). However, ethylene glycol could additionally cause a shift in some segments of luciferase and make it less compact, leading to higher R_g_ and SASA.

(ii) Do the cosolvents change the dynamics of the luciferase structure?

**Figure 8:**
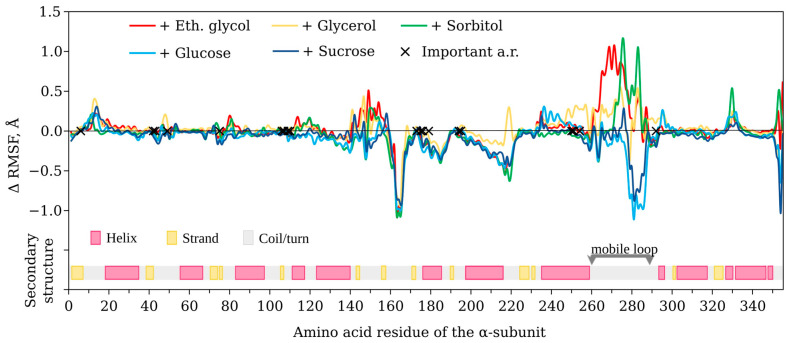
The difference in the root-mean-square fluctuation (RMSF) of C_α_-atoms of *P. leiognathi* luciferase (catalytic α-subunit) in the presence of cosolvents compared to water (ΔRMSF = RMSF_cos_ − RMSF_water_). The area of the functionally important mobile loop is indicated by gray arrows. The crosses refer to the a.r. positions, which are known to be important for luciferase functionality [28]. Secondary structure map shows helixes (pink), strands (yellow), and coils/turns (gray) within the enzyme structure after molecular dynamics simulations in water. The mixtures of water with ethylene glycol (30 wt %), glycerol (40 wt %), sorbitol (40 wt %), glucose (40 wt %), and sucrose (40 wt %) were studied.

The change in the root-mean-square fluctuation (RMSF) of luciferase C_α_-atoms was analyzed. We calculated ΔRMSF = RMSF_cos_ − RMSF_water_, where RMSF_cos_ and RMSF_water_ were parameters in the presence and absence of a cosolvent, respectively. The most pronounced effects were obtained for the 140–300 a.r. range of the catalytic α-subunit of luciferase (Figure 8). All cosolvents decreased RMSF in the 160–170 a.r. range (ΔRMSF < 0). However, the functional role of this segment is not clear yet. The influence of the cosolvents on the dynamics of the functionally important mobile loop (261–290 a.r.) could also be seen. The polyhydric alcohols tended to increase the mobility of this loop (ΔRMSF > 0), while saccharides had the opposite effect (ΔRMSF < 0).

(iii) Do the cosolvents affect the hydration shell of luciferase?

**Figure 9:**
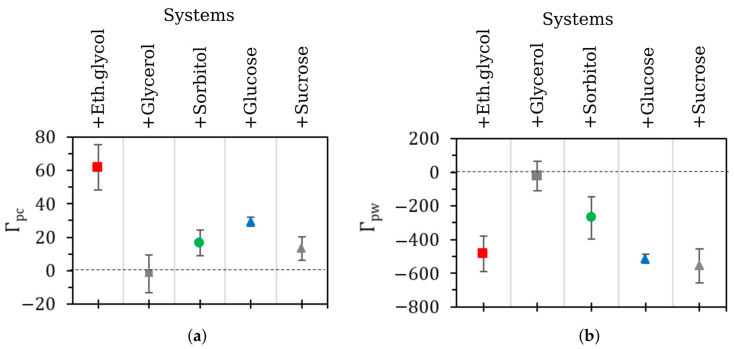
Preferential interaction coefficients for *P. leiognathi* luciferase with cosolvents (**a**) and water (**b**) in the models with the mixtures of water with ethylene glycol (30 wt %), glycerol (40 wt %), sorbitol (40 wt %), glucose (40 wt %), and sucrose (40 wt %). The mean ± standard deviation of three independent runs is shown. The dashed lines refer to the zero level of the coefficients.

Using MD-trajectories, we calculated the minimum-distance distribution function (MDDF) of cosolvent and water molecules relative to luciferase surface [29] in order to estimate the corresponding preferential interaction coefficients Γ (Figure 9). Γ > 0 means that concentration of the molecules near the protein surface is higher than in bulk solution and, vice versa, Γ < 0 refers to the case when the molecules are excluded from the protein surface [30]. The data shown in Figure 9 indicate that, for the majority of the cosolvents, the preferential binding with luciferase surface was observed. Water molecules were excluded from the surface, which was most pronounced in the treatments with ethylene glycol, glucose, and sucrose.

(iv) Do the cosolvents penetrate the active site of luciferase?

**Figure 10:**
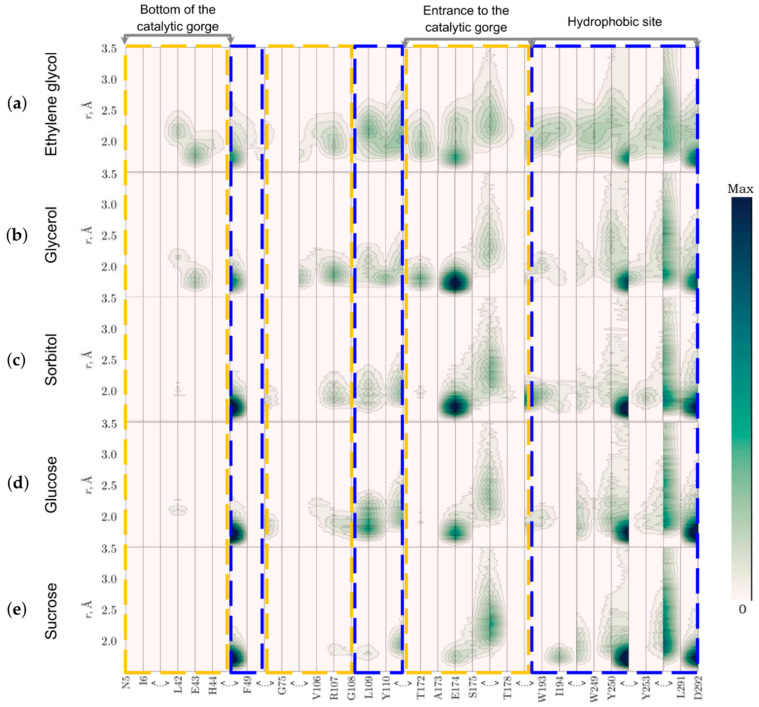
Two-dimensional representation of the minimum-distance distribution function (MDDF) of cosolvent molecules relative to amino acid residues of the *P. leiognathi* luciferase active site indicated at the bottom: (**a**) ethylene glycol 30 wt %; (**b**) glycerol 40 wt%; (**c**) sorbitol 40 wt %; (**d**) glucose 40 wt %; (**e**) sucrose 40 wt %. The dashed frames refer to the binding sites of flavin (yellow) and aldehyde (blue). Gray arrows indicate the elements of the catalytic gorge architecture. The intensity of the green color indicates the probability of cosolvent appearance at the distance r from the residue. <…> means a break in the sequence.

The distribution of the cosolvent molecules within the first hydration shell of the active site of luciferase was analyzed in terms of two-dimensional MDDF maps (Figure 10). It was revealed that only ethylene glycol and glycerol could reach the bottom of the catalytic gorge of luciferase and locate at a distance of <2 Å from the Glu43. Generally, we found that the smaller cosolvent molecule, the higher the probability of its location near the flavin binding site (for example near Arg107), even if the amino acid is at the entrance to the catalytic gorge (Glu174). However, the interaction with hydrophobic site was more probable for larger molecules such as sorbitol, glucose, and sucrose (Phe49, Tyr250). Leu291 and Asp292, which are adjacent to the luciferase mobile loop segment, had a close contact with larger cosolvent molecules as well.

**Figure 11:**
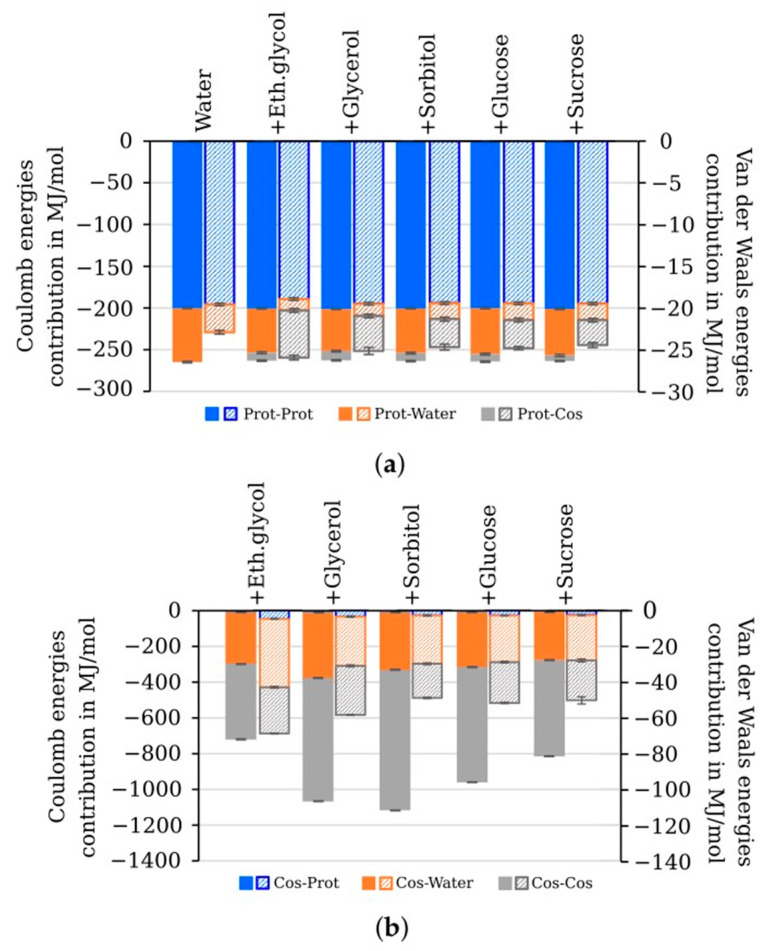
Nonbonded interaction energies in model systems with different cosolvents: (**a**) of *P. leiognathi* luciferase: intramolecular (Prot-Prot), with water (Prot-Water), and cosolvent (Prot-Cos); (**b**) of cosolvent molecules: with protein (Cos-Prot), with water (Cos-Water), and cosolvent (Cos-Cos). Coulomb (filled bars, to the left ordinate) and van der Waals (dashed bars, to the right ordinate) interaction energies are shown. For the models with the mixtures of water with ethylene glycol (30 wt %), glycerol (40 wt %), sorbitol (40 wt %), glucose (40 wt %), and sucrose (40 wt %), the mean ± standard deviation of three independent runs are shown.

Additionally, we calculated nonbonded interaction energies between the components of the studied systems (protein, water, and cosolvent molecules). The major contribution to the Coulomb interaction energies of luciferase was made by intraprotein interactions, while the cosolvents partially replaced water molecules interacting with the protein without substantial changes in the total Coulomb energy (Figure 11a, filled bars). At the same time, the total van der Waals interaction energy of the studied systems was slightly enhanced in the presence of the cosolvents due to protein–cosolvent interaction (Figure 11a, dashed bars). In addition, it was revealed that intraprotein van der Waals interactions became lower in the presence of ethylene glycol (18.87 ± 0.17 MJ/mol) compared with the water environment (19.51 ± 0.16 MJ/mol). That was consistent with the higher R_g_ and SASA values, which indicated less compact protein structure than in the other modeled systems (Figure 7e,f). For cosolvent molecules, the main contributions to the nonbonded interaction energy were made by Coulomb interaction within the cosolvent–water and cosolvent–cosolvent groups. In Figure 11b, the energies of protein–cosolvent interactions can barely be seen compared with those energies. Of all cosolvents, ethylene glycol demonstrated the highest energy of van der Waals interactions and the lowest energy of Coulomb interactions.

### 2.4. Interactions of the Cosolvents with Bacterial Luciferase as Demonstrated by Fluorescence Spectroscopy

**Figure 12:**
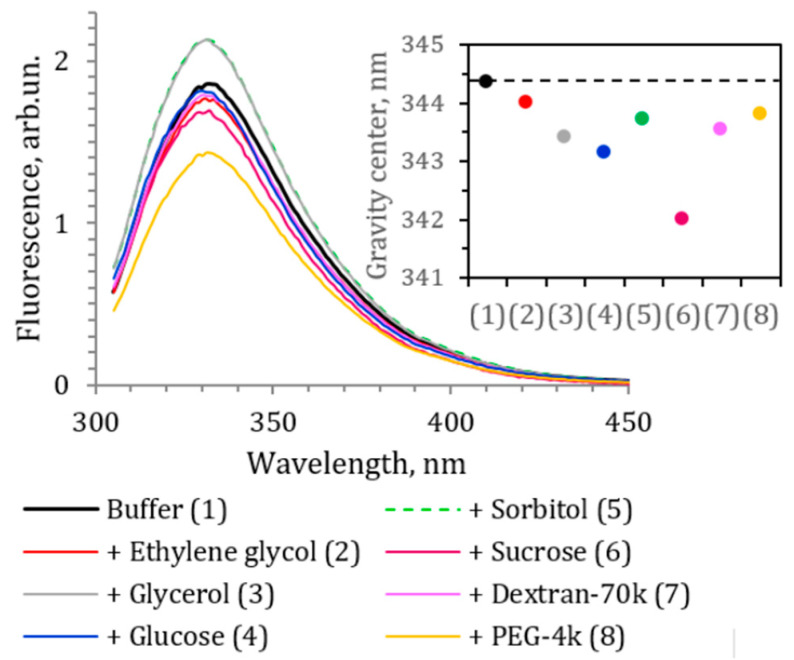
Fluorescence spectra of *P. leiognathi* luciferase under excitation at 295 nm in buffer and in the presence of ethylene glycol (40 wt %), glycerol (40 wt %), glucose (40 wt %), sorbitol (40 wt %), sucrose (40 wt %), dextran (15 wt %), and polyethylene glycol (15 wt %). The insert demonstrates the change in the gravity center of the spectrum. Dashed horizontal line in the insert refers to the position of the gravity center in buffer (344.4 nm). Luciferase concentration in the samples was 1.5 µM. The experimental error of the gravity center value was <0.4 nm.

Structural changes in bacterial luciferase in the presence of cosolvents or intermolecular interaction of the cosolvents and protein surface could be detected by fluorescence spectroscopy technique. The luciferase molecule contains seven tryptophan residues and under excitation at 270–290 nm demonstrates pronounced fluorescent signal, which is quite sensitive to the protein microenvironment and structural changes [26]. We measured the fluorescence spectra of the luciferase in the presence of maximum concentrations of the cosolvents to detect the direct cosolvent–protein interactions or perturbation of the tertiary structure of the protein. Excitation at 295 nm was used, and, thus, tryptophan residues were the major fluorophores of the protein. Figure 12 demonstrates the obtained spectra of luciferase and the position of their gravity center. The cosolvents shifted the fluorescence spectrum to the shorter wavelength. That hypsochromic shift was most pronounced for the sucrose solution (by 2.4 nm), while ethylene glycol had the weakest effect (by 0.3 nm). Additionally, the increase in fluorescence intensity was observed for glycerol and sorbitol solutions, and the decrease for the polyethylene glycol solution. The results indicated that tryptophan residues of luciferase moved to a less polar environment in the presence of the cosolvents, and no sign of protein denaturation (which makes tryptophans more accessible for water) was observed. We incubated luciferase in cosolvent solutions overnight and then repeated spectral measurements, which did not show any change in protein fluorescence spectra compared to the spectra demonstrated in Figure 12. Thus, we excluded the slow denaturation of the protein in the presence of cosolvents as a mechanism of the effects of the media on the luciferase reaction rate.

Previously, we found that among tryptophan residues of *P. leiognathi* luciferase, the most exposed to the solvent were αTrp249 and αTrp276 [26]. They could be responsible for the hypsochromic shift in the protein fluorescence. Moreover, these tryptophans are located in functionally important luciferase segments, mobile loop (αTrp276) and aldehyde binding site (αTrp249), suggesting that protein–cosolvent interactions influencing tryptophan fluorescence could affect the luciferase function too.

Thus, the fluorescence spectroscopy of bacterial luciferase confirms interaction of the cosolvents with the protein surface, which could occur close to the active site of the enzyme.

## 3. Discussion: Distinctions between the Effects of Cosolvents

Aiming to determine the mechanisms of action of different cosolvents on complex multistep biochemical processes, we studied the kinetics of the bioluminescent reaction catalyzed by bacterial luciferase in the presence of polyhydric alcohols and saccharides. Additionally, we applied molecular dynamics simulations to reveal the contribution of protein structure change and protein–cosolvent interactions to kinetic effects. The mathematical modeling of the non-steady-state kinetics of the reaction provided the basis for separating the rates of diffusion-dependent stages and analyzing “pure” catalytic constant of the enzyme.

In our previous studies, we described in detail the effects of the viscous media with glycerol and sucrose on the function of bacterial luciferase [17,18,19]. After expanding the range of viscogenic cosolvents to seven substances of different molecular weights and sizes, we can confidently conclude that some stages of the reaction, both “light” and “dark”, are diffusion controlled at the tested concentrations of the reactants. Namely, the decay of the peroxyflavin intermediate (with the rate constant k_dd_) and the flavin binding (with the rate constant k_1_) demonstrate power-law dependence on viscosity of the medium. The distinctions between the effects of cosolvents are evident in the index of dependence δ: the media with the smaller cosolvents such as ethylene glycol and glycerol cause overdamped effects on k_1_ (δ ≈ 1.8, Figure 6a). That could be the result of the penetration of these molecules into the active site gorge and their presence near the flavin-binding site (Figure 10). Additionally, we recently demonstrated that glycerol tends to interact with the phosphate group of nucleotides, but sucrose does not [31]. Hence, the slowdown of k_1_ to a greater extent than diffusion control implies that it could also result from an increased hydrodynamic volume of flavin in the presence of ethylene glycol and glycerol.

The chemical reaction of oxidation of reduced flavin by molecular oxygen was also found to slow down when viscosity increased, regardless of the type of the cosolvent used (Figure 5a). The deviation of the δ from unity for k_d_(η) could be attributed to the fact that it is a complex (multi-stage and autocatalytic) process [20], and k_d_ in this instance is not a true rate constant, but an apparent one. A similar decrease in the oxidation rate in viscous media was demonstrated for another important cellular metabolite, NADH [32]. As a consequence, the authors suggested that the accumulation of various viscogenic cosolvents in bacterial cells is a mechanism for the preservation of such a low-molecular-weight metabolite.

Interestingly, the uniform dependence k_d_ (η) could be used to estimate the effective viscosity of the solutions with unknown properties. Thus, based on our results presented in Figure 5a, we suppose that the viscosity of PEG-4k solutions (5–15 wt %) determined using a CCVJ molecular rotor was overestimated by 1–2 cP. It is well known that polymeric molecules are unable to form true solutions and it is impossible to characterize their mixtures via a constant value of viscosity [33]. If we accept the correction of the viscosity of PEG-4k solutions in accordance with Figure 5a, viscosity dependencies for this cosolvent in Figure 4 and Figure 6 are very similar to ones obtained for glycerol. At the same time, the viscosity for the second crowding agent Dextran-70k, which was also obtained with a molecular rotor, seems to correspond to the expected value. This could be attributed to the different shapes of the macromolecules, which results in their dissimilar effects on the translational diffusion [34].

Diffusion control of the peroxyflavin intermediate decay (with the rate constant k_dd_) was first demonstrated for media with glycerol and sucrose in our previous work [17]. In the current study, we obtained similar results for glucose and sorbitol solutions (Figure 5b). k_dd_ is a first-order rate constant, and the diffusion limitation of such processes is a rare but not unique event, in which the rate-limiting step is product release [35]. That viscosity dependence of k_dd_ is observed only in the presence of low-molecular-weight cosolvents, but not crowding agents. This suggests that diffusion control requires higher viscosity immediately near the active site, where low-molecular-weight cosolvents can be present, but crowding agents of large size cannot. Intermediates of the biochemical reactions containing C(4a)-(hydro)peroxyflavin adduct are found to adopt many important functions in both prokaryotes and eukaryotes, not only in the bioluminescent system of bacteria [36]. Therefore, the stabilization of such intermediates by a viscous microenvironment could also be one of the biological mechanisms of adaptation of living cells to adverse conditions.

Thus, on the one hand, higher viscosity contributes to the preservation of the substrate and intermediate of the reaction catalyzed by bacterial luciferase, which should increase the bioluminescence efficiency. On the other hand, diffusion limitation decreases the rates of substrate binding as well. In total, it leads to maintaining an approximately constant amount of the emitted quanta in the single-turnover assay in media with viscosity up to 6 cP (Figure 4c).

Ethylene glycol appeared to be a special case among cosolvents used, as is evident from the kinetic (Figure 4, Figure 5b and Figure 6c), structural (Figure 7 and Figure 8), and energetic (Figure 11) parameters. It demonstrated the highest level of preferential interaction with luciferase surface (Figure 9), which could result in the reorientation of the side chains of amino acids, an increase in gyration radius and SASA of the protein (Figure 7e,f, respectively), and a slight reduction in the energy of intraprotein van der Waals interactions (Figure 11a). The fact that the kinetic effects of ethylene glycol are associated with its action on the enzyme, and not on the flavin substrate, is confirmed by the dependence k_d_ (η), which is the same as for other cosolvents (Figure 5a). However, we cannot exclude some interaction of ethylene glycol with decanal, since a strong effect was observed for the association constant of this substrate (Figure 6c). Our findings are consistent with previously reported data demonstrating that ethylene glycol preferably accumulates around the hydrophobic residues of the proteins, which distinguishes it from other cosolvents of polyol type and leads to the unusual effects on protein stability [37]. Different actions of ethylene glycol and glycerol on enzyme kinetics were demonstrated for glucoamylase [38]. The authors suggested that two competing effects appeared in the presence of ethylene-glycol-based cosolvents: first, specific interaction of the cosolvents with the enzyme, which facilitates the product release step, and second, the increase in the solution viscosity, which may inhibit the ability of the enzyme to undergo conformational changes, slowing down the product release step. In the presence of glycerol, only the second mechanism takes place.

Crowding agents PEG-4k and Dextran-70k deserve special attention because their influence on the bioluminescent reaction of bacteria was analyzed for the first time in the present work. Despite the fact that the viscosity of media with crowding agent is a difficult parameter to determine and it depends on many factors [34], for some kinetic parameters of the reaction, we obtained satisfactory results for PEG-4k and Dextran-70k solutions, which were consistent with the diffusion limitation concept (Figure 5a and Figure 6a). However, we have not yet been able to identify a specific influence caused by the effect of the excluded volume on bacterial luciferase functioning. Although it is well known that large oligomeric proteins are the most sensitive to the effects of macromolecular crowding [1], there are examples of the change in the catalytic constant of enzymes comparable in size with bacterial luciferase in the presence of crowding agents [1,6].

**Figure 13:**
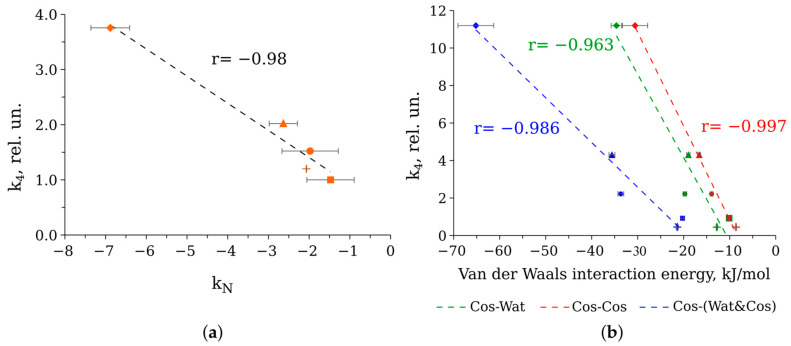
(**a**) Correlation plot between catalytic constant k_4_ of bacterial luciferase in solutions with water activity about 0.98 (ethylene glycol 10 wt %, glycerol 10 wt %, sorbitol 20 wt %, glucose 20 wt %, and sucrose 30 wt %) and Norrish constant k_N_ of the cosolvents used. The Norrish constants were calculated as mean ± standard deviation using the literature data [41]; (**b**) correlation plot between catalytic constant k_4_ of bacterial luciferase in solutions with maximum cosolvent contents (ethylene glycol 30 wt %, glycerol 40 wt %, sorbitol 40 wt %, glucose 40 wt %, sucrose 40 wt %) and van der Waals interaction energies of the cosolvent with water (Cos-Wat, green markers) and itself (Cos-Cos, red markers), as well as the sum of these energies (Cos-(Wat&Cos), blue markers). The energies are calculated per one cosolvent molecule as mean ± standard deviation of three independent runs. The coefficients of linear correlations r are indicated. Dashed lines are linear approximations of the data. Cosolvent markers: ethylene glycol—cross, glycerol—square, sorbitol—circle, glucose—triangle, and sucrose—diamond.

One of the key questions that we sought to answer in our study is which property of the microenvironment determines the catalytic constant (k_4_) of bacterial luciferase. Figure 6d demonstrates that if we remove the influence of diffusion-limited stages, we see that the efficiency of luciferase catalysis depends on the type of the cosolvent rather than on the viscosity of the medium. Analysis of various properties of the viscous solutions used led us to assume that the water–cosolvent interaction pattern could modulate luciferase catalytic constant. Namely, we found a correlation between k_4_ at water activity in the solutions a_w_ = 0.98 and Norrish constants k_N_ of the cosolvents (Figure 13a). k_N_ characterizes the ability of the substance to change water activity after dissolution [39]. In brief, if k_N_ of a substance is >0, then water activity in its solution is higher than the water molar fraction and vice versa (see a review on water activity concept in [40,41,42,43]). We were limited by the set of the cosolvent concentrations, which were used to vary the viscosity of the media and could hardly analyze correlations at different a_w_. Further study is required to reveal the influence of the media with finely fitted water activity characteristics on the reaction kinetics. Using calculated interaction energies of Coulomb and van der Waals types for model systems with the maximum cosolvent concentrations, we searched for their correlation with k_4_ values under these conditions. The best correlations were found for Van der Waals energies of cosolvent–water and cosolvent–cosolvent interactions calculated per one molecule of the cosolvent (Figure 13b). We think these results are consistent with the correlation obtained for Norrish constants reported in a number of other studies (Figure 13a).

Several examples can be found among the literature data relating enzymatic catalysis to water activity [44]. To thoroughly study this relation for bacterial luciferase, further research is needed. It was proposed for some enzymes that the presence of water molecules in their active sites lowered the transition state to accelerate the reaction kinetics [45]. Such enzymes are expected to be sensitive to the presence of cosolvents in reaction media and to the differences in the cosolvent–water interactions.

## 4. Materials and Methods

### 4.1. Chemicals

Flavin mononucleotide (FMN) was purchased from Sigma-Aldrich (St. Louis, MO, USA). EDTA (ROTH) was used as an electron donor for FMN photoreduction. Lyophilized recombinant luciferase *Photobacterium leiognathi* (99% purity) was purchased from “Prikladnye Biosistemy Ltd.” (Krasnoyarsk, Russia). Reactants were dissolved in a potassium phosphate buffer (0.05 M, pH 6.9). The concentrations of FMN and luciferase were determined spectrophotometrically with the extinction coefficients ε_445_ = 12,200 M^−1^ cm^−1^ and ε_280_ = 80,000 M^−1^ cm^−1^, respectively. The stock solution of decanal (Acros Organics, Fair Lawn, NJ, USA) in ethanol with 2 × 10^−3^ M concentration was used freshly prepared. For viscous media, we used mixtures of the buffer with ethylene glycol (Komponent-Reaktiv, Moscow, Russia), glycerol (Panreac), sorbitol (Panreac), glucose (Reachim, Moscow, Russia), and sucrose (Panreac) with a mass fraction of cosolvent of 10–40% and dextran of the average molecular weight 70 kDa (Dia-M, Moscow, Russia) and polyethylene glycol of the average molecular weight 4 kDa (Panreac) with a mass fraction of cosolvent of 5–15%.

### 4.2. Measurements of the Kinetics of the Bioluminescent Reaction and Dark Decay Processes

Bacterial luciferase reaction was analyzed using a single-turnover assay [19]. The reaction was initiated by mixing two solutions using a stopped-flow technique by SX-20 (Applied Photophysics, Leatherhead, UK). The first solution consisted of 3 × 10^−5^ M FMN, 1 × 10^−2^ M EDTA, and a small volume of decanal. Before adding aldehyde, the solution was bubbled with argon for 10 min. The second solution was an air-equilibrated buffer containing 2 × 10^−6^ M of bacterial luciferase. FMN was reduced photochemically by keeping the first solution in the light of an incandescent lamp for 10 min. Bioluminescence kinetics was measured at 20 °C for 15 s. Each kinetic curve was obtained by averaging at least 5 repetitions.

The kinetics of reduced flavin autoxidation was obtained by recording the absorbance change at 445 nm after mixing an anaerobic solution of reduced FMN (30 μM) with the air-equilibrated buffer. The kinetics of Intermediate II decay was obtained by recording the absorbance change at 380 nm after mixing an anaerobic solution of luciferase (5 μM) and reduced flavin (30 μM) with the air-equilibrated buffer. In both cases, the buffer solutions in the treatments also contained viscous cosolvents. This procedure was described elsewhere [17].

For all experiments in viscous media, the mixtures of the buffer with ethylene glycol, sorbitol, glucose (10, 20, 30, 40 wt %), dextran (5, 10, 12.5, 15 wt %), or polyethylene glycol (5, 10, 15, 20 wt %) were used instead of the buffer.

### 4.3. Simulation of Kinetics of the Bioluminescent Reaction

The kinetics of light emission in bioluminescent reaction (Figure 1) was formalized using a system of nonlinear ordinary differential equations and numerically solved using the Scilab program developed at the Laboratory of Theoretical Biophysics, the Institute of Biophysics SB RAS (Krasnoyarsk, Russia). The algorithm searched for the theoretical value of the intensity at each time step that most closely matched the experimental data. To do this, the most suitable values of the rate constants were selected, while the least-squares fit error was used as a minimization parameter. Two decay constants (k_d_, k_dd_) were fixed during simulation because they were obtained in specially conducted experiments. Bioluminescence kinetic curves obtained at five different concentrations of decanal (10, 20, 30, 40, and 50 μM) in the buffer and in the presence of cosolvents were used as input data and simultaneously simulated. As an output, a set of individual kinetic constants was obtained for each medium. The relative error of each simulation did not exceed 4.2%. The simulation procedure is described in detail elsewhere [19].

### 4.4. Kinetics Parameters Analysis

Bioluminescence kinetics curves were used to determine empirical parameters: the peak intensity (I_max_) and the total quantum yield (Q*) as the maximum signal and an area under the kinetic curve, respectively. The initial velocity of the reaction (v_0_) was evaluated as the slope of the starting linear part of the kinetic curve. Lastly, the final part of the kinetic curve was approximated using an exponential function, and the decay constant (k_decay_) was calculated as an indicator of the function. The process of calculating these parameters is described in detail elsewhere [19]. The viscosity values of the buffer–cosolvent mixtures at 20 °C were taken from the published data (ethylene glycol [46], glycerol [46], sorbitol [47], glucose [48], sucrose [46]). For mixtures of the buffer with dextran and polyethylene glycol, the viscosity was determined via fluorescent technique using molecular rotor 9-(2-carboxy-2-cyanovinyl)julolidine (CCVJ) as described in [49].

The dependences of the rate constants on the solution viscosity were fitted via a power function using Origin 8.0 software (OriginLab).

### 4.5. Molecular Dynamics of Bacterial Luciferase in the Presence of Cosolvents

The all-atom molecular dynamics (MD) of bacterial luciferase surrounded by water and cosolvents was simulated using GROMACS 2020.04 with CHARMM36 force field [50,51]. In order to model specific interactions of *P. leiognathi* luciferase surface and functional loops with cosolvents, its initial coordinates were obtained via homology-based modeling as described in [27]. Luciferase was modeled without any of the reaction components and their derivatives, since their structures in complex with the enzyme have not been crystalized yet (except for the product of the reaction [52]), and conformational changes in the protein were the focus of this study.

We conducted three independent 100 ns molecular dynamics simulations for each system; the parameters and modeling routine can be found elsewhere [27]. In all simulations, the temperature was kept constant at T = 300 K. The numbers of cosolvent/water molecules in model boxes with cosolvents were: ethylene glycol (30 wt %)—3694/28,546, glycerol (40 wt %)—3320/25,958, sorbitol (40 wt %)—1678/27,419, glucose (40 wt %)—1697/28,241, and sucrose (40 wt %)—893/28,968. The topology of the cosolvents was defined using the CHARMM Small Molecule Library [51]. Analysis of the luciferase structural stability and the nonbonded interaction energies between the components of the system was performed using GROMACS plugins. Open source software Complex-Mixtures.jl, implemented in Julia language, was used to compute protein–cosolvent minimum-distance distribution functions (MDDF) and preferential interaction coefficients for cosolvent molecules (Γ_pc_) and for water molecules (Γ_pw_) [29].

### 4.6. Fluorescence Spectroscopy of Bacterial Luciferase in Viscous Media

The spectral characteristics of luciferase at a concentration of 1.5 µM were measured at maximal concentrations of cosolvents. The measurements were carried out at 20 °C after incubation for at least 5 min.

The absorption spectra were measured using a Cary 5000 spectrophotometer (Agilent Technologies, Mulgrave, Australia) with an integrated Pelletier temperature controller. Steady-state fluorescence spectra of the protein were measured with a Fluorolog-3 spectrofluorometer (Horiba Jobin Yvon, Edison, NJ, USA). The intensity was collected within the 305–450 nm range under excitation at 295 nm. All fluorescence spectra were corrected for PMT spectral sensitivity, inner filter effect, and background signal [53].

The spectral shift in the fluorescence was analyzed in terms of the gravity center (GC) of the spectrum, which was determined using the following equation:

$$GC=\frac{\sum_{\lambda =305}^{450}I_{\lambda }\lambda \;}{\sum_{\lambda =305}^{450}I_{\lambda }\;},$$

where *λ* is the wavelength and *I_λ_* is the emission intensity at that given wavelength.

## 5. Conclusions

In the current work, we studied the influence of media with cosolvents of different molecular sizes on the elementary stages of the bioluminescent reaction catalyzed by bacterial luciferase. We found that the increasing viscosity of the media slows down some reaction stages, both bimolecular and monomolecular, in a diffusion-control manner, with distinctions attributed to the specific interactions of cosolvents with the enzyme. However, the catalytic constant of the enzyme appears to be viscosity independent, indicating that no large domain movements of luciferase occur during the catalytic act. The correlation of the catalysis rate of luciferase with parameters describing interactions between water and cosolvent molecules (Norrish constant, van der Waals interaction energies) was obtained for the first time.

The influence of the cosolvents on water activity and the change in viscosity of the medium are generally two sides of the same coin. They both are results of water–cosolvent interaction. That is why it seems impossible to find the cosolvent with, for example, a Norrish constant similar to that of sucrose but with low viscogenic action. However, in the majority of the investigations devoted to the effects of media on biochemical processes, only one side is discussed. Meanwhile, in vivo metabolic processes have to adapt to the conditions where both diffusion of the reactants, especially those with large molecular size, is limited and water activity is significantly reduced. Such conditions arise, in particular, when cells begin to accumulate special small molecules in response to adverse factors such as high or low temperature, salinity, pressure, and others [54]. The case study of the reaction catalyzed by bacterial luciferase demonstrated that luminous bacteria could develop some kind of compensation for kinetics slowdown via a more effective catalytic act, to maintain the stable level of light emission under varying environmental conditions.

We suppose that our findings could be extended to enzymes with similar chemistry (other flavin-dependent monooxygenases [55]) rather than with similar function (other luciferases), because structures of luciferins and luciferases vary significantly in different organisms. However, based on the participation of oxygen in all bioluminescent reactions, we could assume that its binding is not diffusion controlled for other luciferases either.

## Data Availability

The data presented in this study are available on request from the corresponding author.

## Supplementary Materials

The following supporting information can be downloaded at: https://www.mdpi.com/article/10.3390/life13061384/s1, Figure S1: The structural parameters change of *P. leiognathi* luciferase obtained during 100-ns MD simulation using the models with water and the mixtures with (1) ethylene glycol (30 wt %), (2) glycerol (40 wt %), (3) sorbitol (40 wt %), (4) glucose (40 wt %), and (5) sucrose (40 wt %).
