# Supplementary material for: The Role of Cosolvent–Water Interactions in Effects of the Media on Functionality of Enzymes: A Case Study of Photobacterium leiognathi Luciferase
Source: Life (Basel). 2023 Jun 13;13(6):1384. doi: 10.3390/life13061384 (PMC10301326; doi:10.3390/life13061384)
Supplement: Supplementary file 1 [file life-13-01384-s001.zip › life-2343982-supplementary.pdf]

The role of cosolvent–water interactions in effects of the media on functionality of enzymes: a case study of *Photobacterium leiognathi* luciferase

Albert E. Lisitsa [1,], Lev A. Sukovatyi [1], Anna A. Deeva [1], Dmitry V. Gulnov [1], Elena N. Esimbekova [1,2], Valentina A. Kratasyuk [1,2], and Elena V. Nemtseva [1,2]

[1]Siberian Federal University, 660041, Svobodny 79, Krasnoyarsk, Russia
[2]Institute of Biophysics SB RAS, 660036, Akademgorodok 50/50, Krasnoyarsk, Russia

Supplementary Material

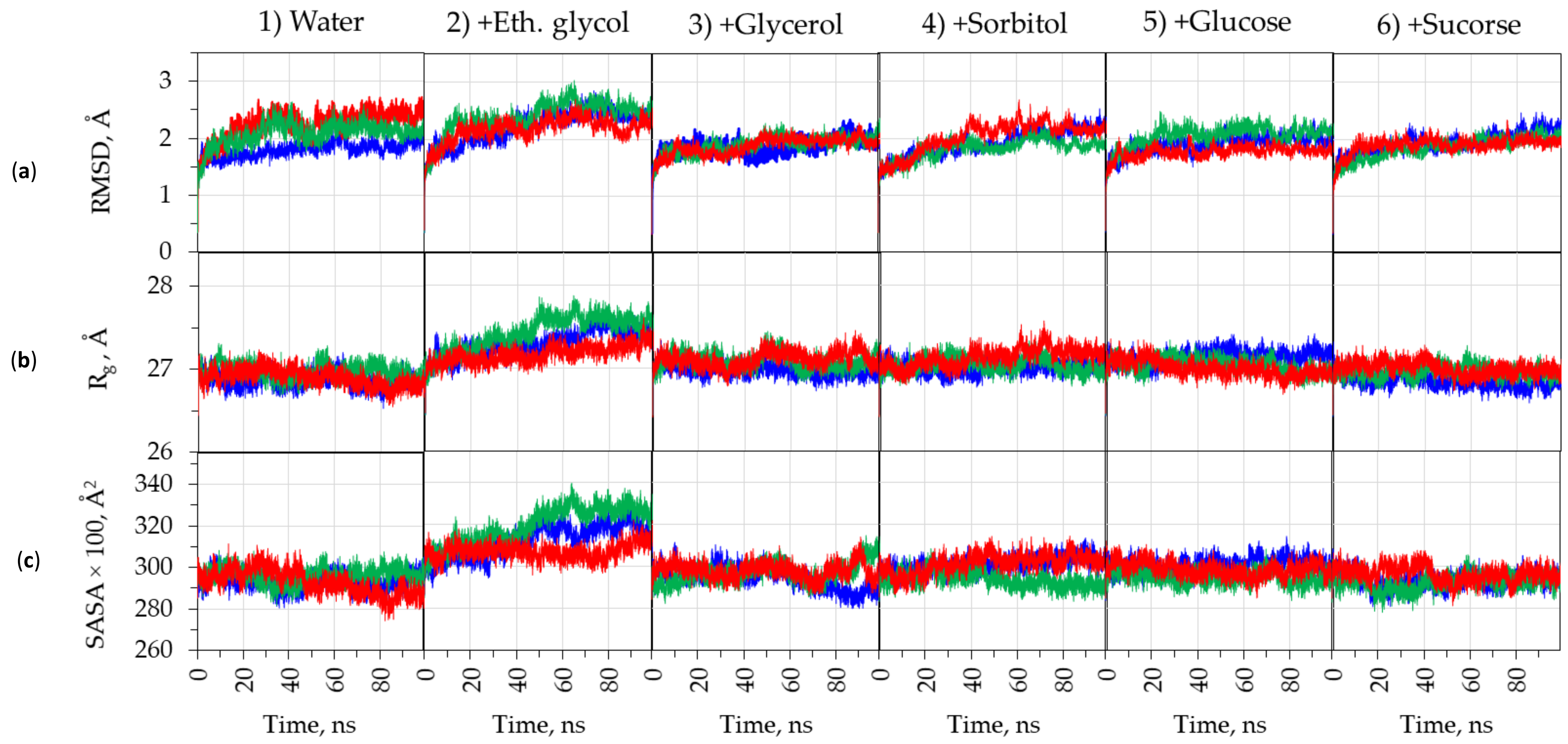

Figure S1. The structural parameters change of *P. leiognathi* luciferase obtained during 100-ns MD simulation using the models with water and the mixtures with 1) ethylene glycol (30 wt %), 2) glycerol (40 wt %), 3) sorbitol (40 wt %), 4) glucose (40 wt %), and 5) sucrose (40 wt %): (**a**) the root-mean-square-deviation of C_α-atoms (RMSD); (**b**) the gyration radius for all protein atoms (R_g); (**c**) the total solvent accessible surface area (SASA). Three independent runs are shown by blue, green and red curves.
